# Macrophage-Specific MCPIP1/Regnase-1 Attenuates Kidney Ischemia-Reperfusion Injury by Shaping the Local Inflammatory Response and Tissue Regeneration

**DOI:** 10.3390/cells11030397

**Published:** 2022-01-24

**Authors:** Andrea Ribeiro, Ewelina Dobosz, Moritz Krill, Paulina Köhler, Marta Wadowska, Stefanie Steiger, Christoph Schmaderer, Joanna Koziel, Maciej Lech

**Affiliations:** 1 LMU Klinikum, Medizinische Klinik und Poliklinik IV, Ludwig-Maximilians-Universität Munich, 80336 Munich, Germany; andrea.sof@gmail.com (A.R.); moritz.krill@juakrill.de (M.K.); koehlerpaulina@gmail.com (P.K.); stefanie.steiger@med.uni-muenchen.de (S.S.); 2 Department of Nephrology, Klinikum Rechts der Isar, Technical University Munich, 80336 Munich, Germany; christoph.schmaderer@mri.tum.de; 3 Department of Microbiology, Faculty of Biochemistry, Biophysics and Biotechnology, Jagiellonian University, 30-387 Krakow, Poland; e.dobosz@uj.edu.pl (E.D.); marta.wadowska@doctoral.uj.edu.pl (M.W.); joanna.koziel@uj.edu.pl (J.K.)

**Keywords:** MCPIP1, inflammation, macrophages, ischemia-reperfusion

## Abstract

Sterile inflammation either resolves the initial insult or leads to tissue damage. Kidney ischemia/reperfusion injury (IRI) is associated with neutrophilic infiltration, enhanced production of inflammatory mediators, accumulation of necrotic cells and tissue remodeling. Macrophage-dependent microenvironmental changes orchestrate many features of the immune response and tissue regeneration. The activation status of macrophages is influenced by extracellular signals, the duration and intensity of the stimulation, as well as various regulatory molecules. The role of macrophage-derived monocyte chemoattractant protein-induced protein 1 (MCPIP1), also known as Regnase-1, in kidney ischemia-reperfusion injury (IRI) and recovery from sterile inflammation remains unresolved. In this study, we showed that macrophage-specific *Mcpip1* deletion significantly affects the kidney phenotype. Macrophage-specific *Mcpip1* transgenic mice displayed enhanced inflammation and loss of the tubular compartment upon IRI. We showed that MCPIP1 modulates sterile inflammation by negative regulation of *Irf4* expression and accumulation of IRF4+ cells in the tissue and, consequently, suppresses the post-ischemic kidney immune response. Thus, we identified MCPIP1 as an important molecular sentinel of immune homeostasis in experimental acute kidney injury (AKI) and renal fibrosis.

## 1. Introduction

The immune system is a central component orchestrating the pathophysiology of various acute forms of kidney injury (AKI) and plays a crucial role in the progression of chronic kidney disease (CKD). Moreover, a dysregulated immune system can directly trigger immune-mediated kidney disease or influence the course of injury [1]. This may take place during, e.g., uncontrolled inflammatory responses or phenotypic changes, within immune cell populations. Ischemia is a common clinical event that may occur in the kidney due to a sudden drop in blood flow to the organ. It leads to high morbidity and mortality, but is also involved in the development of CKD, as well as the progression from pre-existing CKD to end-stage kidney disease (ESKD) [2,3]. A complex network of interactions between renal parenchymal cells, resident and recruited immune cells sets major innate immunity pathways in motion by activating pattern recognition receptors and releasing various inflammatory mediators [4,5,6]. Among cells involved in renal inflammation, neutrophils (recruited in the acute phase), large numbers of Ly-6C+ (inflammatory) monocytes and resident macrophages are cell populations, which initiate inflammatory processes that, in return, influence acute inflammation, regeneration, or pro-fibrotic changes within the tissue [7,8,9,10]. Under basal conditions, tissue-resident macrophages maintain homeostasis. They show remarkable plasticity that allows them to change the phenotype, orchestrate the response to injury, and repair [11]. For instance, heart macrophages are necessary for proper electrical conduction [12]. In the gut, these cells interact with enteric neurons to maintain peristalsis [13]. In the kidney, macrophages were described to regulate the resolution of injury (including ischemia-related damage) and progression to fibrosis [14]. They were shown to promote tissue-environmental changes that can cause irreversible tissue damage followed by loss of integrity and function [9].

Studies show that the mononuclear phagocytic system significantly affects the pathophysiology of ischemic AKI [15]. Macrophages, which to a great extent orchestrate the resolution of initial inflammation and the tissue rearrangement, display a crucial factor that links pro- and anti-inflammatory pathways involved in post-ischemic tissue conditions [16]. The complex mechanisms of immune dysregulation leading to organ injury, followed by the resolution of inflammation, are far-reaching and not well understood. Among candidates that play a crucial role in regulating inflammatory responses and homeostasis is MCPIP1 [17]. Monocyte chemotactic protein-1-induced protein-1 (MCPIP1), also known as Regnase-1 (Regulatory RNase 1) or ZC3H12A, was shown to recognize stem-loops in RNA [18]. This unique feature allows rapid regulations of transcripts in response to micro-environmental changes, which promotes cellular adaptation and maintains homeostasis [19,20,21]. MCPIP1 functions as deubiquitinase and displays an RNase catalytic center [22,23,24,25]. In 2009, the protein was described for the first time as a ribonuclease, which binds the 3′UTR of the IL-6 gene and degrades IL-6 mRNA [26]. To date, various transcripts have been identified as targets for MCPIP1 [22,27]. The expression of MCPIP1 is induced by various factors such as MCP1, TNFα, and IL-1β but also during viral, bacterial, and fungal infections [19,28,29,30,31,32]. Our recent study showed that depletion of MCPIP1 in macrophages and granulocytes is sufficient to trigger a severe autoimmune disease that was characterized by the expansion of B cells and plasma cells and the spontaneous production of autoantibodies, including anti-dsDNA, anti-Smith, and anti-histone antibodies [33]. Moreover, we showed that MCPIP-1-dependent degradation of anti-apoptotic transcripts regulates spontaneous apoptosis of primary neutrophils [34].

In the present study, we aimed to examine whether granulocyte/macrophage cell-specific (LysM+) dysregulation of homeostasis (using cell-specific knockout of MCPIP1) participates in the process of ischemic kidney injury and inflammation. We hypothesized that macrophage- and granulocyte-specific MCPIP1 is a molecular sentinel of immune homeostasis in experimental kidney disease and suppresses post-ischemic kidney inflammation.

## 2. Materials and Methods

Animal studies: Cell-specific knockout of the Mcpip-1 gene in myelomonocytic cells was achieved by crossing floxed MCPIP-1 mice (Zc3h12a^tm1c(EUCOMM)Hmgu^, MGI:6115370) with LysM-Cre mice (Jackson Laboratory, Bar Harbor, ME, USA). Female and male mice were housed in sterile filter top cages with a 12-h dark/light cycle. The study was carried out following the principles of the Directive 2010/63/EU on the Protection of Animals Used for Scientific Purpose and with the approval of the local government authorities (II LKE in Krakow and Regierung von Oberbayern). The ischemia-reperfusion injury was performed in 12–16-week-old, age-matched control (either Flox or Lys-M-Cre mice) and gene-deficient female mice. Contralateral kidneys served as intra-individual controls. Groups of mice (*n* = 5–9) were anesthetized before the left kidney pedicles were clamped for 30 min with a microaneurysm clamp via 1 cm flank incisions (Medicon, Tuttlingen, Germany). Body temperature was maintained at 35–37 °C throughout the procedure. Mice were sacrificed either 3 or 30 days after surgery, to investigate short and long-term consequences of kidney injury.

Kidney parameters: Urinary Lipocalin-2/NGAL levels were determined using a commercially available mouse ELISA DuoSet kit (R&D Systems, Park Abingdon, UK) according to the manufacturer’s recommendations. Mouse urine samples were diluted in serial dilutions ranging from 1:10^3^ to 1:10^6^. Creatinine levels in urine were determined using the creatinine assay DiaSys kit (Diagnostic Systems GmbH, Holzheim, Germany). Urine samples were prepared in dilutions of 1:10.

Evaluation of kidney histopathology: Kidneys were fixed in 4% buffered formalin, processed overnight (dehydration, clearing) in a tissue processor, and embedded in paraffin. We used the indicated antibodies and followed the recommendation of the manufacturer: TNF alpha antibody, cat. orb371962, Biorbyt, 1:600 overnight at 4 °C (incl. blocking with 10% Goat Serum); IRF-4 Antibody (3E4), cat. sc-130921, Santa Cruz Biotechnology, 1:50 overnight at 4 °C; F4/80 Antibody (CI-A3-1), NB600-404 Novus Biologicals, 1:100 overnight at 4 °C; Lotus Tetragonolobus Lectin (LTL), Biotinylated, cat. B-1325, Vector Laboratories, 1:2000 for 1 h at RT; Picro-Sirius Red solution 0.1%, cat. 365548, Sigma-Aldrich. For quantitative histological analysis, we used Adobe Photoshop CS4Extended and examined the percentage of stained high power fields (6–9 mice per group). We used an average of 4 hpf representing the cortex (including glomeruli and proximal tubules) of each kidney.

In vitro Studies: primary mouse tubular epithelial cells (pTECs) were seeded (5 × 10^5^ cells/mL) in 10% FCS 1% PS K1 medium in six-well plates and grown to 50% confluence. TECs were isolated according to an established protocol. In brief, the kidney capsule was peeled off and the kidneys were minced finely with the back of a syringe and digested with Collagenase D (working concentration 1.5 mg/mL) for 30 min at 37 °C. The digested kidneys were sieved through a 70 µm filter and centrifuged at 1500 rpm for 5 min at 4 °C, with a brake. The pellet was re-suspended in 2 mL PBS and layered very carefully on 10 mL of 31% Percoll and centrifuged at 3000 rpm for 10 min at 4 °C, without brake. Cells were washed twice with PBS and tubular epithelial cells were grown from proximal tubular segments cultured in K1 medium composed by Dulbeccos’s Modified Eagles’s Medium supplied with 1 M Hepes (pH 7.55), 10% FCS, Hormone mix (HBSS, 31.25 pg/mL PGE-1, 3.4 pg/mL T3, 18 ng/mL Hydrocortisone), 9.6 µg/mL ITSS, 20 ng/mL EGF and 1% PS. The medium was changed two to three days after isolation. BMDM: bone marrow was isolated from the femur and tibia. An 18 G needle was pushed through the bottom of a 0.5 mL Eppendorf tube and put into a 1.5 mL Eppendorf tube. The bones were placed into the 0.5 mL Eppendorf tube and centrifuged at 10,000 rpm, 4 °C for 15 s. The pellet was re-suspended in 1 mL of 0.155 M NH_4_Cl (RBC lysis buffer at room temperature) by slowly pipetting and 2 mL more were added. The mixture was kept at room temperature for 1 min. The reaction was stopped by diluting the lysis buffer with medium (10–20 mL) followed by centrifugation at 1500 rpm, 4 °C for 2 min. Cells were washed with medium and centrifuged again under the same conditions. The cell suspension was passed through a cell strainer (70 μm) and centrifuged again. The pellet was re-suspended in a 1 mL medium and cells were counted. Cells were seeded in 12/6 well plates (1.5 × 10⁶/12 well plate or 3 × 10⁶/6 well plate) in 1 or 2 mL of Dulbeccos’s Modified Eagles’s Medium supplied with 10% FCS (or mouse serum), 1% PS, and rmM-CSF at a concentration of 2 ng/mL, respectively. After 2/3 days, 1/2 mL of medium with rmM-CSF was added to the seeded cells. On day 5, the medium was removed and replaced by a fresh medium supplied with rmM-CSF. On day 7, cells were ready for stimulation. All recombinant cytokines were obtained from ImmunoTools. To identify the effect of hypoxia on the primary renal tubular cells and macrophages, experiments were conducted in a hypoxic incubator with oxygen control containing 1% O_2_, 5% CO_2_, and 94% N_2_. Cells were cultured in 1% FCS for 48 h. Immortalized J774 were grown in a 75 cm^2^ flask in Dulbeccos’s Modified Eagles’s Medium containing 10% FCS and 1% PS. Subcultures were prepared by scraping. For the 75 cm^2^ flasks all but 10 mL of the culture medium was removed. Cells were dislodged from the flask with a cell scraper, aspirated, and dispensed into new flasks, in a ratio of 1:3 to 1:6. The medium was replaced two or three times a week. All in-vitro experiments were performed a minimum of two independent times.

Flow Cytometry: intracellular cytokine staining was performed using the BD Cytofix Cytoperm kit. J774 cells were cultured in RPMI 1640 5% FCS and 1% PS media until they reached confluence. Cells were then centrifuged and the pellet suspended in complete media. For the transfection with small interfering RNA (siRNA), one million cells were mixed in an opti-MEM medium with a mix of Lipofectamine 2000 (ThermoFisher Scientific, Waltham, MA, USA, #11668019) and control-scrambled siRNA (80 nM) or *Mcpip1* siRNA (80 nM), following the instructions provided by the company. Then, 24 h post-transfection, cells were stimulated for 4 h (for qRT-PCR) or 24 h (flow cytometry) as indicated in the figures. Macrophages were analyzed by flow cytometry using a BD FACSCanto II flow cytometer and FlowJo v8.7 software (BD Biosciences, Heidelberg, Germany). Differentiated macrophages were characterized using the surface markers indicated in the figures. All antibodies were obtained from BioLegend. Fixable viability dye efluor780 (ebioscience, San Diego, CA, USA) was used in every sample to identify dead cells.

Real-time quantitative PCR: SYBR Green Dye detection system was used for quantitative real-time PCR on Light Cycler 480 (Roche, Mannheim, Germany). Gene-specific primers (225 nM, Metabion, Martinsried, Germany) were used. Standard controls for genomic DNA contamination, RNA quality, and general PCR performance were included. The data were evaluated using the 2ΔCT method. RNA was isolated from the samples (equal cell count, equal amount of tissue) using the Norgen Biotek Total RNA Purification kit (Thorold, ON, Canada, #37500) and MagNA Lyser Green beads (Roche, Basel, Switzerland) according to the manufacturer’s instructions. Total RNA was measured using a NanoDrop ND-1000 Spectrophotometer (ThermoFisher Scientific). For cDNA conversion, 1 µg of total RNA was used. A mixture of 5× Buffer (ThermoFisher Scientific, supplement of SuperScript II Reverse Transcriptase), 0.1 M DTT (ThermoFisher Scientific, supplement of SuperScript II Reverse Transcriptase), 25 mM dNTPs (ThermoFisher Scientific, #R0186), 40 U/µL rRNasin (Promega, Madison, WI, USA, #N2518), 10× Hexanucleotide Mix (Roche, #11277081001), 15 µg/mL Linear Acrylamid (ThermoFisher Scientific, #AM9520) and 10,000 U SuperScript II Reverse Transcriptase (ThermoFisher Scientific, #18064016) was added to the RNA and incubated in a Thermomixer Comfort (Eppendorf) at 42 °C for 90 min. To inactivate the reverse transcriptase, samples were incubated again for 5 min at 90 °C. At the end of the process, samples were stored at −20 °C. We used the following primer sequences (5′–3′): gapdh (fw: catggccttccgtgttccta, rv: cctgcttcaccaccttctca); il18 (fw: ccaaatcagttcctcttggc, rv: ggccaaagttgtctgattcc); il13 (fw: cacacaagaccagactcccc, rv: tctgggtcctgtagatggca); il10 (fw: acagccgggaagacaataact, rv: cctgcattaaggagtcggtta); il4 (fw: atggatgtgccaaacgtcct, rv: agcttatcgatgaatccaggca); vimentin (fw: agagagaggaagccgaaagc, rv: tccactttccgttcaaggtc); ccl2 (fw: gctacaagaggatcaccagca, rv: gtctggacccattccttcttg); ccl17 (fw: tgcttctggggacttttctg, rv: ataggaatggcccctttgaa); tnfa (fw: agggtctgggccatagaact, rv: ccaccacgctcttctgtctac); pcna (fw: tggataaagaagaggaggcg, rv: ggagacagtggagtggcttt); cxcl10 (fw: ggctggtcacctttcagaag, rv: atggatggacagcagagagc); ctgf (fw: agctgacctggaggaaaaca, rv: ccgcagaacttagccctgta); col4a (fw: gtctggcttctgctgctctt, rv: cacattttccacagccagag); ifng (fw: aggaactggcaaaaggatgg, rv: tcattgaatgcttggcgctg); irf4 (fw: tgcaagctctttgacacaca, rv: caaagcacagagtcacctgg); tgfb (fw: ggagagccctggataccaac, rv: caacccaggtccttcctaaa); ccl5 (fw: ccacttcttctctgggttgg, rv: gtgcccacgtcaaggagtat); il6 (fw: tgatgcacttgcagaaaaca, rv: accagaggaaattttcaataggc); inos (fw: ttctgtgctgtcccagtgag, rv: tgaagaaaaccccttgtgct); il12 (fw: tcttctcaccgtgcacatcc, rv: tggccaaactgaggtggttt); hprt (fw: ctggtgaaaaggacctctcgaag, rv: ccagtttcactaatgacacaaacg); mcpip-1 (fw: cctgtggtcatcgacggaag, rv: gaaggatgtgctggtctgtgata); kim1 (fw: tggttgccttccgtgtctct, rv: tcagctcgggaatgcacaa); fsp1: (fw: cagcacttcctctctcttgg, rv: tttgtggaaggtggacacaa); asma (fw: actgggacgacatggaaaag, rv: gttcagtggtgcctctgtca); e-cadherin (fw: gaggtctacaccttcccggt, rv: ccactttgaatcgggagtct); egf (fw: actggtgtgacaccaagaggtc, rv: ccacaggtgatcctcaaacacg); vegf (fw: ctgctgtaacgatgaagccctg, rv: gctgtaggaagctcatctctcc).

Statistical analysis: Data were expressed as mean ± SD. The Mann–Whitney U test was used for direct comparisons between single groups i.e., wild type and knockout cells/mice due to small sample size and non-parametric distribution of data (D’Agostino-Pearson normality test). We used GraphPad Prism software. The one-way analysis of variance (ANOVA) with post hoc Tukey was used if more than two independent groups were compared. A *p*-value < 0.05 indicated statistical significance. Statistical significance was indicated as follows: *p*-value of <0.05 (*); *p*-value of <0.01 (**); *p*-value of <0.001 (***).

## 3. Results

### 3.1. Mcpip1 Is Induced in Macrophages and Renal Tubular Cells In Vitro following Oxidative Stress

**Figure 1:**
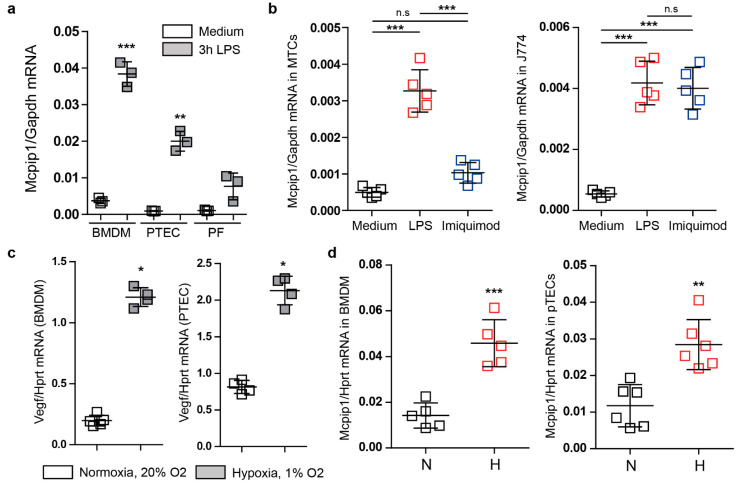
(**a**) The mRNA expression levels of *Mcpip1* in bone marrow-derived macrophages (BMDM), primary tubular epithelial cells (PTEC), and primary fibroblasts (PF) upon stimulation with LPS (100 ng/mL) for 3 h. (**b**) The mRNA expression levels of *Mcpip1* in a tubular epithelial cell line (MTC) and J774 macrophage cell line upon LPS (100 ng/mL) and Imiquimod (1 µg/mL) stimulation for 3 h. (**c**) The mRNA expression levels of *Vegf* in bone marrow-derived macrophages (BMDM) and primary tubular epithelial cells (PTEC) upon 48-h culture under normal oxygen (N) and hypoxic (H) conditions. (**d**) The mRNA expression levels of *Mcpip1* in bone marrow-derived macrophages (BMDM) and primary tubular epithelial cells (PTEC) upon normal levels of oxygen (N) and hypoxic conditions (H). Data are shown as means ± SD. * *p* < 0.05. ** *p* < 0.01. *** *p* < 0.001.

Recent reports have indicated that MCPIP1 is involved in ischemic injury [35]. In this study, we used primary renal parenchymal cells, macrophages, and J774 macrophages to investigate the effects of exposure to reduced oxygen levels. We detected a low basal mRNA expression under resting conditions in macrophages from wild-type mice. We observed almost no basal expression of Mcpip1 in tubular cells and fibroblasts from wild-type mice (Figure 1a). However, upon stimulation of TLR4 signaling, which mediates kidney reperfusion injury [36], we observed a potent induction of Mcpip1 expression in primary cells (Figure 1a). Similarly, cell lines stimulated with LPS (TLR4) showed an induction of the Mcpip1 transcript 3 h following ligand challenge. We used imiquimod (TLR7) as a control agent and, as expected, we observed the induction of Mcpip1 expression in a macrophage cell line that expresses significant mRNA levels of TLR7 (Figure 1b) [37]. Next, we used primary bone marrow-derived macrophages (BMDM) and primary tubular epithelial cells to study the effects of hypoxia and oxidative stress on Mcpip1 expression (Figure 1c). We used the vascular endothelial growth factor Vegf as an indicator of hypoxic conditions (Figure 1c). Hypoxia is a well-described time-dependent and robust regulator of Vegf expression and Hprt1 is a well-characterized hypoxia-independent housekeeping gene [38]. The increased expression of Mcpip1 in both primary cell types supports a hypothesis that MCPIP1 might play a significant role in ischemic injury and hypoxia-mediated signaling events (Figure 1d). These data show that MCPIP1 is not only expressed in immune cells but also parenchymal cells of the kidney. Thus, MCPIP1 signaling due to hypoxia may be involved in protection from oxidative stress.

### 3.2. Mcpip1 Is Induced in the Kidney Tissue following IRI and Macrophage Expression of Mcpip1 Regulates Acute Inflammation upon Kidney Ischemic Damage

**Figure 2:**
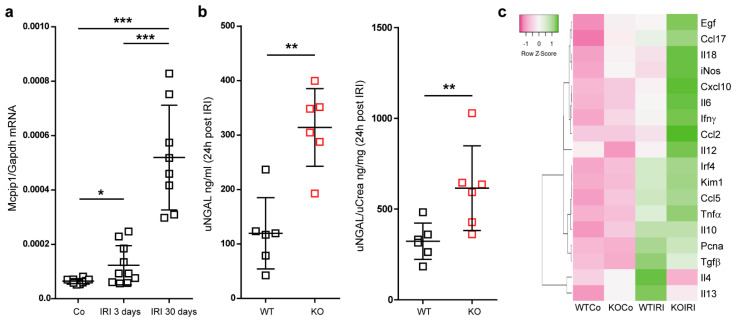
(**a**) The mRNA expression levels of *Mcpip1* in wild-type kidneys upon IRI (control, 3 days and 30 days). (**b**) The concentration of urinary NGAL and ratio uNGAL/Crea in wild-type and knockout mice (KO) upon acute IRI (24 h post-IRI). (**c**) The expression level of selected genes in the kidney upon acute IRI (3 days post-IRI). A heat map shows altered genes from expression analysis of pre-selected transcripts. Genes indicated in green are upregulated and genes indicated in pink are downregulated to highlight differences between the samples. The rows are Z-Score scaled. The information about a single gene expression across the samples (controls and IRI in wild-type *n* = 6 and knockout kidneys *n* = 6) is given but the expression levels of gene X to gene Y cannot be concluded. Data are shown as means ± SD. * *p* < 0.05. ** *p* < 0.01. *** *p* < 0.001.

In line with various studies, MCPIP1 negatively regulates TLR-mediated inflammation and may significantly affect the course of ischemic injury followed by regeneration [39,40]. We, therefore, induced unilateral renal IRI for 30 min in wild-type and cell-specific (LysM) *Mcpip1* knockouts to assess kidney outcomes after 3 and 30 days upon injury (early and chronic stage of injury). Based on the *Mcpip1* expression at the early and late time point of tissue damage and its role in innate immune signaling, we hypothesized that MCPIP1 could affect not only acute but also chronic kidney inflammation and tissue remodeling (Figure 2a). To investigate the effects of macrophage-specific deletion of MCPIP1 in IRI, we evaluated kidney parameters, which are variable in unilateral injury. Ischemic injury in macrophage-specific MCPIP1-knockouts resulted in significantly higher urinary neutrophil gelatinase-associated lipocalin levels, uNGAL (Figure 2b). As expected, there was no significant difference in serum creatinine and BUN levels between the groups (unilateral injury, data not shown). We observed increased expression of pro-inflammatory mediators in the kidneys of macrophage-specific MCPIP1-knockouts 3 days upon IRI (Figure 2c). The above results demonstrate that macrophage expression of *Mcpip1* protects against IRI. Thus, lack of MCPIP1 in macrophages aggravates acute kidney inflammation and, based on *Mcpip1* expression level, has an impact on long-term outcomes following IRI.

### 3.3. Macrophage MCPIP1 Promotes Resolution of the Chronic Inflammatory Response Associated with Aggravated Kidney Injury and Fibrosis after IRI

**Figure 3:**
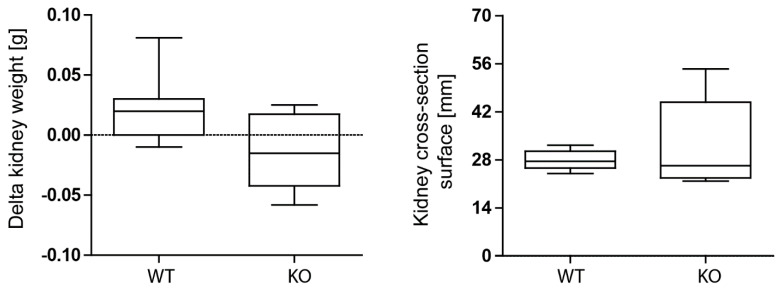
No differences in kidney delta weight (weight of contralateral kidney—weight of ischemia-reperfusion kidney) and kidney size between wild-type (WT; *n* = 9) and *Mcpip1*-knockout (KO; *n* = 6) mice 30 days after IRI.

Next, we induced unilateral kidney IRI for 30 min in wild-type and macrophage-specific *Mcpip1*-knockout mice to assess kidney outcomes after 30 days. As expected, we observed recovery of wild-type kidneys. However, kidneys of macrophage-specific MCPIP1-deficient mice did not display the typical loss of kidney weight that we expected based on our previous data investigating other anti-inflammatory factors upon IRI [41,42]. Hypertrophy/tissue rearrangement of the ischemic kidney 30 days after acute IRI was estimated by delta kidney weight (contralateral kidney weight—ischemic kidney weight) and kidney cross-section surface (Figure 3).

**Figure 4:**
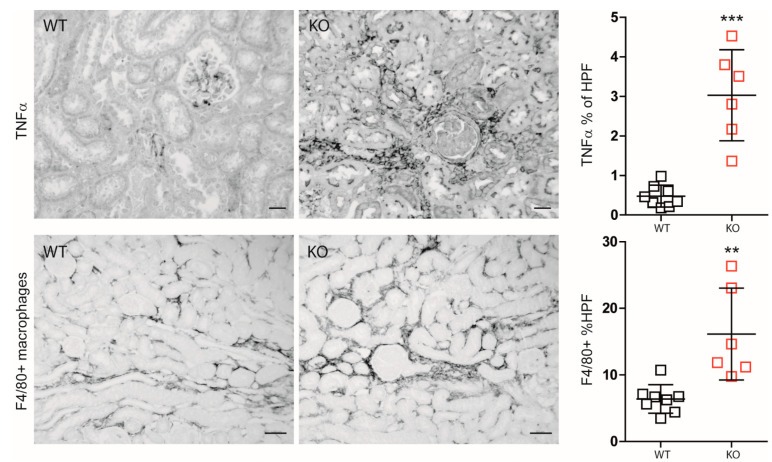
Differences between wild-type (WT; *n* = 8) and *Mcpip1*-knockout (KO; *n* = 6) mice in TNF-α renal staining and F4/80+ macrophages 30 days upon ischemic injury. Scale bar = 50 μm. Data are shown as means ± SD. ** *p* < 0.01. *** *p* < 0.001.

**Figure 5:**
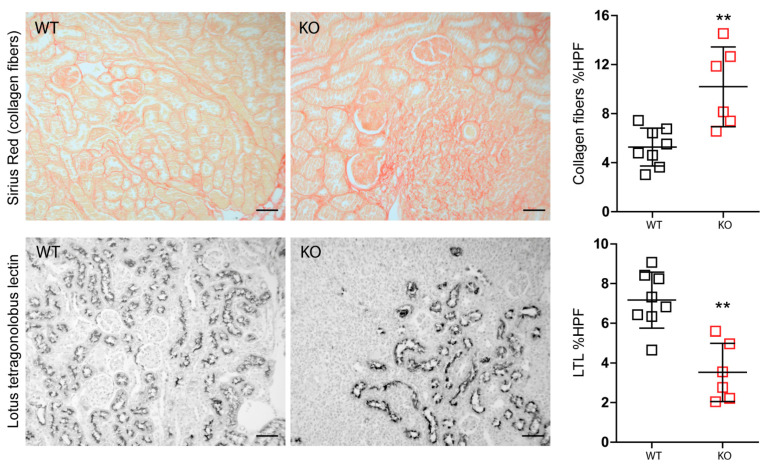
Differences between wild-type (*n* = 8) and *Mcpip1*-knockout mice (*n* = 6) in fibrosis and proximal tubular epithelial cells 30 days upon ischemic injury. Scale bar = 50 μm. Data are shown as means ± SD. ** *p* < 0.01.

Nevertheless, IRI in macrophage-specific *Mcpip1*-knockout mice resulted in increased inflammation as indicated by TNF-α staining and infiltration of F4/80+ macrophages as compared to wild-type mice (Figure 4). In addition, *Mcpip1*-knockout mice showed a significantly increased inflammatory response that was associated with increased fibrosis (Figure 5 upper panel) and loss of tubular cell mass (Figure 5 lower panel), as evidenced by LTL staining.

**Figure 6:**
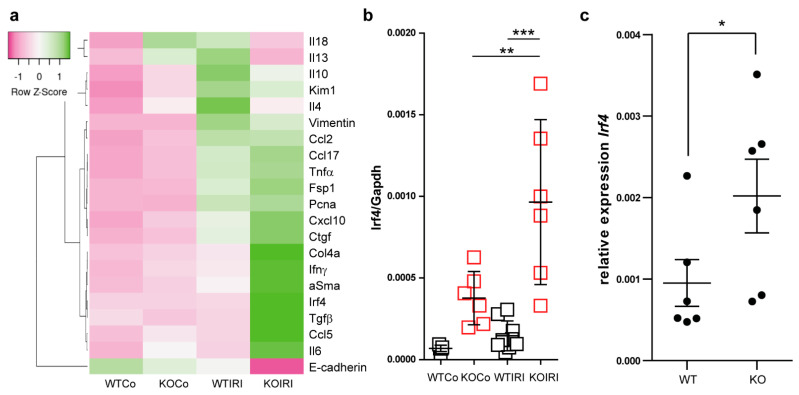
(**a**) The expression level of selected genes in the kidney upon chronic kidney injury (30 days post-IRI). The heat map shows the expression analysis of pre-selected transcripts. Genes indicated in green are upregulated and genes indicated in pink are downregulated to highlight differences between the samples. The rows are Z-score scaled (controls and IRI in wild-type (WT; *n* = 9) and knockout kidneys (*n* = 6)) (**b**) mRNA expression levels of *Irf4* in kidneys from WT and *Mcpip1*-knockout mice 30 days after IRI (control and 30 days upon injury). (**c**) BMDM isolated from *Mcpip1*-knockout mice express significantly higher levels of *Irf4*. Data are shown as means ± SD. * *p* < 0.05. ** *p* < 0.01. *** *p* < 0.001.

Consistently, we observed significantly increased mRNA expression of fibrosis markers including *Ctgf*, *collagen4a*, and *Tgf-β*, *αSMA*, and *Fsp1* in post-ischemic kidneys from macrophage-specific *Mcpip1*-knockout mice as compared to wild-type mice (Figure 6a). Furthermore, intrarenal E-cadherin mRNA expression indicating epithelial regeneration was decreased in *Mcpip1*-knockout mice (Figure 6a). In addition, our results revealed that IRF4 was highly expressed in *Mcpip1*-knockout mice upon injury as compared to wild-type mice (Figure 6b). Similar results were obtained in BMDMs (Figure 6c). Thus, the lack of MCPIP1 also aggravates chronic renal inflammation, promotes loss of tubular epithelial cells, and fibrosis. Moreover, *Mcpip1* seems to significantly regulate *Irf4* transcript, a transcription factor that is important for alternative-macrophage activation [43].

### 3.4. MCPIP1 Regulates the Number of IRF4+ Cells in the Kidney Postischemic Injury

**Figure 7:**
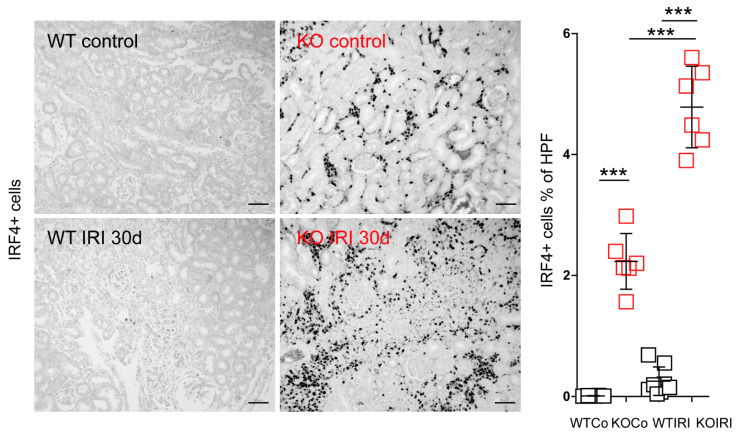
Kidney sections from wild-type (WT; *n* = 8) and *Mcpip1*-knockout (KO; *n* = 6) mice were stained for IRF4 and the percentage of IRF4+ cells was quantified 30 days after IRI. Scale bar = 50 μm. Data are shown as means ± SD. *** *p* < 0.001.

Polarization of macrophages to proinflammatory or alternatively activated cells is important for mounting inflammatory responses and tissue rearrangement, respectively. IRF4 has been identified as one key transcription factor that controls alternative (M2) macrophage polarization [43]. To examine further the effect of macrophage-specific *Mcpip1*-deletion on *Irf4* expression, we evaluated the infiltration of IRF4+ cells into the kidney upon IRI (Figure 7). Surprisingly, we observed only a slight increase in the number of IRF4+ cells in wild-type mice upon IRI, unlike previously published data [42]. This could be explained by the relatively mild IRI procedure used in the present study (30 min of ischemia followed by reperfusion). As IRF4 was described as one key transcription factor in the development and function of innate and adaptive immune cells [43,44,45], the number of IRF4+ cells will only partially represent macrophages. However, it is important to mention that macrophages are the predominant cell population found in the interstitium during chronic inflammation. Moreover, studies using IRF4-deficient immune cells showed a reduced migratory capacity of these cells [46]. Therefore, high intracellular *Irf4* levels could lead to increased migratory potential and macrophage infiltration in *Mcpip1*-knockout mice.

### 3.5. MCPIP1 Modulates the Macrophage Phenotype upon Inflammation

**Figure 8:**
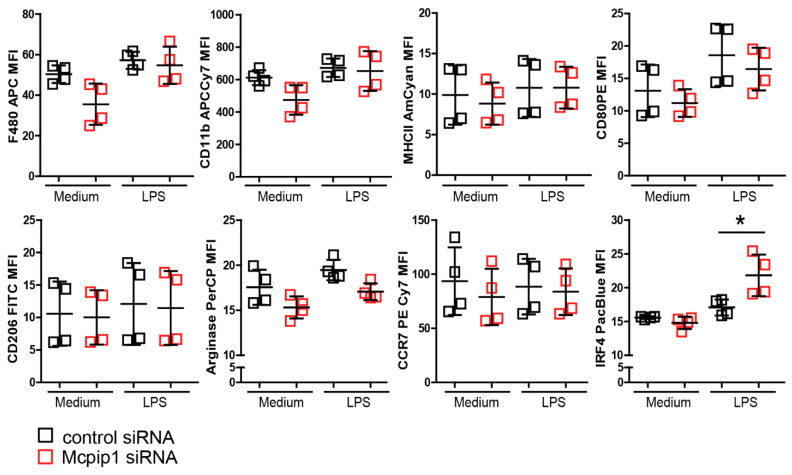
MCPIP1 inhibited LPS-stimulated IRF4 expression. J774 cells were stimulated with LPS (10 ng/mL) for 24 h. Mean fluorescence intensity of M1/M2 macrophage marker representing protein level was detected by flow cytometry. Data are shown as means ± SD. * *p* < 0.05.

Previous studies using human THP1 showed that M2-polarized macrophages are the predominant population present during hypoxia [47]. To look at the effect of hypoxia-modified microenvironments on macrophages in-vitro, we first used primary bone-marrow-derived macrophages. Unfortunately, we failed to differentiate and grow macrophages derived from *Mcpip1*-knockout or knock-down cells under hypoxic conditions (data not shown). Therefore, we used the J774 macrophage cell line and transfected them with scramble or *Mcpip1*-specific siRNA prior to stimulation with LPS for 4 or 24 h (Figure 8). After transfection, total RNAs were extracted. *Mcpip1* mRNA was detected, and the successful knockdown was confirmed by qRT-PCR (data not shown). We found that stimulation of J774 macrophages with a low concentration of LPS (10 ng/mL) for 24 h did not change the status of most M1 and M2 markers (Figure 8). Interestingly, protein expression of IRF4 (one of the main transcription factors for M2 macrophages) was induced in *Mcpip1* knockdown cells upon LPS treatment. Thus, MCPIP1 is involved in macrophage-phenotype shaping during the inflammatory response by limiting the expression of *Irf4*.

## 4. Discussion

Kidney ischemic injury is a considerable complication after a sudden impairment of the blood flow to tissue that could be triggered by various pathophysiological states and medications, as well as organ transplantation [48]. Macrophages contribute to cellular pathophysiology in AKI associated with ischemia as well as to tissue repair processes. Macrophage-dependent resolution of inflammation and tissue rearrangement must be suitably adapted and regulated to avoid fibrosis and CKD. The molecular mechanisms involved in these processes are not yet sufficiently characterized and IRI-induced AKI retains an unresolved clinical challenge despite the myriad efforts. For these reasons, we explored whether the immunomodulatory properties of MCPIP are relevant in the course of AKI and sterile inflammation. MCPIP1 is known to act as a negative regulator of LPS-induced macrophage activation [49] and MCPIP1 deficiency in myeloid cells can contribute to autoimmune and severe inflammatory responses [27,33,34,50], suggesting that MCPIP1 in macrophages and granulocytes orchestrates inflammation and immunity. Our study now confirmed that MCPIP1 negatively regulates sterile inflammation and, thereby, limits acute IRI-induced AKI. Moreover, our data revealed a potential regulatory function of macrophage-specific MCPIP1 during the AKI-to-CKD transition phase, a time where macrophages are present in high numbers. MCPIP1 is important for tissue regeneration because LysM-specific knockout mice presented with loss of tubular epithelial cells and renal fibrosis upon kidney IRI compared to wild-type mice. This is in line with previous studies showing that *Mcpip1* knockdown aggravates liver IRI in mice [35]. Thus, MCPIP1 acts as a negative regulator of immune responses in the kidney [41,42,51,52,53]. Yet, various negative regulators display unique signaling abilities and may function in different cells, processes, and phases of inflammatory response.

Interstitial macrophage signaling is one of the reasons for progressive AKI and the development of fibrosis, numerous drug-based and macrophage/monocyte depletion experimental therapies have been studied [54]. For instance, early depletion of macrophages reduced acute tubular necrosis, inflammation, and fibrosis. However, early therapeutic blockade of initial leukocyte infiltration aggravated post-ischemic kidney injury [55,56,57], while depletion of macrophages at 3 to 5 days after injury slows tubular cell proliferation and repair [58]. Such findings support the hypothesis that controlled inflammatory responses associated with resident cells and immune cell infiltration are crucial for an appropriate model of action upon injury and efficient resolution of inflammation. Considering that different types of macrophages control processes of inflammation, regeneration, and fibrosis during the entire course of injury, it is yet difficult to estimate what therapy and in which phase might be beneficial to prevent AKI. Macrophages, apart from their differentiation status, exhibit a distinct activation status triggered by the sterile microenvironment. Based on surface markers, macrophages have been broadly classified as inflammatory M1 and alternative M2 phenotypes. This polarization is important for regulating the balance between inflammation and tissue repair. However, upon activation macrophages display a more sophisticated multipolar spectrum of gene expression signature and function [59]. Several studies reported that MCPIP1 induced alternative M2 macrophages. Kapoor et al. reported that MCPIP1 promotes IL4-induced M2 polarization and that macrophages from mice with myeloid-targeted overexpression of MCPIP1 differentiated into M2-like macrophages with a reduced response to LPS. Consistent with previous studies, our data showed a shift towards a pro-inflammatory environment in macrophage-specific *Mcpip1*-knockout mice upon injury, suggesting a role for MCPIP1 in the early phase of IRI-induced AKI. In contrast, macrophages from mice with myeloid-specific deletion of MCPIP1 rather differentiate into M1-like macrophages with an enhanced phagocytic activity [60]. The same study reported that MCPIP1 inhibits M1 polarization by blocking NF-κB. However, our in-vitro experiments did not prove that *Mcpip1* knockdown could induce an M1 macrophage phenotype. This might be due to the J774 cell line used in the experiments or the relatively low concentration of LPS that represents physiological local conditions, i.e., *endotoxin* levels in patients with *sepsis* [61]. Despite our efforts, we failed to show a clear macrophage phenotype switch. Interestingly, we found a high upregulation of *Irf4* in ischemic kidneys isolated from mice deficient in MCPIP1 in macrophages, which is consistent with previous data demonstrating that *Irf4* serves as a substrate for MCPIP1 [62]. In addition, Peng et al. showed that *Irf4* mRNA is stabilized in CD4+ T cells from Zc3h12a−/− mice [63]. Thus, the data suggest that MCPIP1 deficiency promotes an alternative pathway of M2 macrophage polarization via IRF4 [43]. Moreover, we previously showed that MCPIP1 is downregulated in neutrophils in the presence of GM-CSF, which prevented neutrophil apoptosis [34]. Thus, MCPIP1 might regulate the GM-CSF/IRF4/CCL17 pathway [64]. The chemokine CCL17, which is involved in inflammation and pain, was found to be induced upon treatment of macrophages with GM-CSF via JMJD3-regulated IRF4 signaling [64,65]. Although CCL17 has been reported to drive a pro-fibrotic macrophage phenotype [66], the lack of GM-CSF or IRF4 can also alter macrophage polarization independent of CCL17 [67]. Thus, the exact mechanism of action remains to be elucidated. However, our results prove that MCPIP1 plays an important role in regulating *Irf4* and the number of IRF4+ cells in the kidney during sterile inflammation. Thus, the pathogenesis of AKI, as well as the development and progression of fibrosis, seem to be the result of a complex interplay between cellular and inflammatory factors. The enhanced infiltration of IRF4+ cells could also explain the relatively normal ischemic kidney size upon 30 days of injury (usually scarred and shrink in size kidneys) [42,68] because we observed primarily inflammatory M1 macrophages in chronically inflamed kidneys of *Irf4*−/− mice [42]. Therefore it is possible that the elevated *Irf4* levels and the high number of IRF4+ cells in *Mcpip1*-knockout mice could partially substitute the environment-dependent effects on macrophage polarization because IRF4 drives macrophages toward an alternatively activated/M2 phenotype [69,70,71]. Recently, Sasaki et al. demonstrated that IRF4 expressed in myeloid cells could promote the development of fibrosis by regulating AKT-mediated monocyte recruitment and phosphatidylinositol 3-kinase/AKT signaling in the injured kidney [72]. This is in contrast to our finding showing enhanced fibrosis in IRF4-deficient mice upon IRI-induced AKI [42]. Both a lack of IRF4+ cells and enhanced numbers of IRF4+ cells in the kidney could potentially lead to tissue damage. An editorial article from 2021 hypothesized that IRF4 might promote cell migration but, at the same time, inhibit macrophage activation [73]. However, further experiments, e.g., phenotypic analysis and migration assays with macrophages overexpressing IRF4 as well as IRF4-deficient cells, are needed to prove this hypothesis.

The lack of quantitative measurement of neutrophil infiltration in an early stage of the injury is a limitation of this study and the role of neutrophils in the pathologies of kidneys observed in our in vivo model cannot be ruled out. A comprehensive analysis of inflammatory events in the first 48 h upon injury could shed light on the effects of neutrophils recruitment during sterile inflammation especially since MCPIP-1 regulates the survival of neutrophils [34]. Our present data suggest also that MCPIP1 plays a role in tubular epithelial cells; this could have a significant impact on the disease. Previously, we showed that the MCPIP-1 protein level was higher in epithelial cells than in myeloid cells. The RNase activity of the protein regulates the transcript of interleukin (IL)-8 and determines the inflammatory responses [27].

In conclusion, MCPIP1 plays a protective role in IRI-induced AKI by regulating inflammation and the number of IRF4+ cells in the tissue. Extensive investigations regarding MCPIP1-dependent mechanisms controlling macrophage phenotype are needed to understand inflammatory responses. MCPIP, in light of its immunosuppressive properties, might serve as a promising therapeutic target in inflammatory diseases.

## Data Availability

Not applicable.

## References

[1] Tecklenborg J., Clayton D., Siebert S., Coley S.M. (2018). The role of the immune system in kidney disease. Clin. Exp. Immunol..

[2] Bucaloiu I.D., Kirchner H.L., Norfolk E.R., Hartle J.E., Perkins R.M. (2012). Increased risk of death and de novo chronic kidney disease following reversible acute kidney injury. Kidney Int..

[3] Hsu R.K., Hsu C.-Y. (2016). The Role of Acute Kidney Injury in Chronic Kidney Disease. Semin. Nephrol..

[4] Tang P.C.-T., Zhang Y.-Y., Chan M.K.-K., Lam W.W.-Y., Chung J.Y.-F., Kang W., To K.-F., Lan H.-Y., Tang P.M.-K. (2020). The Emerging Role of Innate Immunity in Chronic Kidney Diseases. Int. J. Mol. Sci..

[5] Wang Y.-H., Zhang Y.-G. (2017). Kidney and innate immunity. Immunol. Lett..

[6] Anders H.-J., Lech M. (2013). NOD-like and Toll-like receptors or inflammasomes contribute to kidney disease in a canonical and a non-canonical manner. Kidney Int..

[7] Black L.M., Lever J.M., Agarwal A. (2019). Renal Inflammation and Fibrosis: A Double-edged Sword. J. Histochem. Cytochem. Off. J. Histochem. Soc..

[8] Mosser D.M., Hamidzadeh K., Goncalves R. (2021). Macrophages and the maintenance of homeostasis. Cell. Mol. Immunol..

[9] Lech M., Gröbmayr R., Ryu M., Lorenz G., Hartter I., Mulay S.R., Susanti H.E., Kobayashi K.S., Flavell R.A., Anders H.-J. (2014). Macrophage phenotype controls long-term AKI outcomes--kidney regeneration versus atrophy. J. Am. Soc. Nephrol..

[10] Lin S.L., Castaño A.P., Nowlin B.T., Lupher M.L.J., Duffield J.S. (2009). Bone marrow Ly6Chigh monocytes are selectively recruited to injured kidney and differentiate into functionally distinct populations. J. Immunol..

[11] Lech M., Gröbmayr R., Weidenbusch M., Anders H.-J. (2012). Tissues use resident dendritic cells and macrophages to maintain homeostasis and to regain homeostasis upon tissue injury: The immunoregulatory role of changing tissue environments. Mediat. Inflamm..

[12] Hulsmans M., Clauss S., Xiao L., Aguirre A.D., King K.R., Hanley A., Hucker W.J., Wülfers E.M., Seemann G., Courties G. (2017). Macrophages Facilitate Electrical Conduction in the Heart. Cell.

[13] De Schepper S., Verheijden S., Aguilera-Lizarraga J., Viola M.F., Boesmans W., Stakenborg N., Voytyuk I., Schmidt I., Boeckx B., Dierckx de Casterlé I. (2018). Self-Maintaining Gut Macrophages Are Essential for Intestinal Homeostasis. Cell.

[14] Nelson P.J., Rees A.J., Griffin M.D., Hughes J., Kurts C., Duffield J. (2012). The renal mononuclear phagocytic system. J. Am. Soc. Nephrol..

[15] Sharfuddin A.A., Molitoris B.A. (2011). Pathophysiology of ischemic acute kidney injury. Nat. Rev. Nephrol..

[16] Lech M., Anders H.-J. (2013). Macrophages and fibrosis: How resident and infiltrating mononuclear phagocytes orchestrate all phases of tissue injury and repair. Biochim. Biophys. Acta.

[17] Xu J., Fu S., Peng W., Rao Z. (2012). MCP-1-induced protein-1, an immune regulator. Protein Cell.

[18] Leppek K., Schott J., Reitter S., Poetz F., Hammond M.C., Stoecklin G. (2013). Roquin promotes constitutive mrna decay via a conserved class of stem-loop recognition motifs. Cell.

[19] Iwasaki H., Takeuchi O., Teraguchi S., Matsushita K., Uehata T., Kuniyoshi K., Satoh T., Saitoh T., Matsushita M., Standley D.M. (2011). The IκB kinase complex regulates the stability of cytokine-encoding mRNA induced by TLR–IL-1R by controlling degradation of regnase-1. Nat. Immunol..

[20] Zhou L., Azfer A., Niu J., Graham S., Choudhury M., Adamski F.M., Younce C., Binkley P.F., Kolattukudy P.E. (2006). Monocyte chemoattractant protein-1 induces a novel transcription factor that causes cardiac myocyte apoptosis and ventricular dysfunction. Circ. Res..

[21] Uehata T., Iwasaki H., Vandenbon A., Matsushita K., Hernandez-Cuellar E., Kuniyoshi K., Satoh T., Mino T., Suzuki Y., Standley D.M. (2013). Malt1-induced cleavage of regnase-1 in CD4^+^ helper T cells regulates immune activation. Cell.

[22] Mino T., Murakawa Y., Fukao A., Vandenbon A., Wessels H.H., Ori D., Uehata T., Tartey S., Akira S., Suzuki Y. (2015). Regnase-1 and roquin regulate a common element in inflammatory mRNAs by spatiotemporally distinct mechanisms. Cell.

[23] Xu J., Peng W., Sun Y., Wang X., Xu Y., Li X., Gao G., Rao Z. (2012). Structural study of MCPIP1 N-terminal conserved domain reveals a PIN-like RNase. Nucleic Acids Res..

[24] Yokogawa M., Tsushima T., Noda N.N., Kumeta H., Enokizono Y., Yamashita K., Standley D.M., Takeuchi O., Akira S., Inagaki F. (2016). Structural basis for the regulation of enzymatic activity of Regnase-1 by domain-domain interactions. Sci. Rep..

[25] Uehata T., Akira S. (2013). MRNA degradation by the endoribonuclease Regnase-1/ZC3H12a/MCPIP-1. Biochim. Biophys. Acta Gene Regul. Mech..

[26] Matsushita K., Takeuchi O., Standley D.M., Kumagai Y., Kawagoe T., Miyake T., Satoh T., Kato H., Tsujimura T., Nakamura H. (2009). Zc3h12a is an RNase essential for controlling immune responses by regulating mRNA decay. Nature.

[27] Dobosz E., Wilamowski M., Lech M., Bugara B., Jura J., Potempa J., Koziel J. (2016). MCPIP-1, Alias Regnase-1, Controls Epithelial Inflammation by Posttranscriptional Regulation of IL-8 Production. J. Innate Immun..

[28] Blazusiak E., Florczyk D., Jura J., Potempa J., Koziel J. (2013). Differential regulation by toll-like receptor agonists reveals that MCPIP1 is the potent regulator of innate immunity in bacterial and viral infections. J. Innate Immun..

[29] Liang J., Wang J., Saad Y., Warble L., Becerra E., Kolattukudy P.E. (2011). Participation of MCP-induced protein 1 in lipopolysaccharide preconditioning-induced ischemic stroke tolerance by regulating the expression of proinflammatory cytokines. J. Neuroinflamm..

[30] Lin R.-J., Chu J.-S., Chien H.-L., Tseng C.-H., Ko P.-C., Mei Y.-Y., Tang W.-C., Kao Y.-T., Cheng H.-Y., Liang Y.-C. (2014). MCPIP1 suppresses hepatitis C virus replication and negatively regulates virus-induced proinflammatory cytokine responses. J. Immunol..

[31] Lim Y.-J., Choi J.-A., Lee J.-H., Choi C.H., Kim H.-J., Song C.-H. (2015). Mycobacterium tuberculosis 38-kDa antigen induces endoplasmic reticulum stress-mediated apoptosis via toll-like receptor 2/4. Apoptosis.

[32] Liu S., Qiu C., Miao R., Zhou J., Lee A., Liu B., Lester S.N., Fu W., Zhu L., Zhang L. (2013). MCPIP1 restricts HIV infection and is rapidly degraded in activated CD4^+^ T cells. Proc. Natl. Acad. Sci. USA.

[33] Dobosz E., Lorenz G., Ribeiro A., Wurf V., Wadowska M., Kotlinowski J., Schmaderer C., Potempa J., Fu M., Koziel J. (2021). Murine myeloid cell MCPIP1 suppresses autoimmunity by regulating B-cell expansion and differentiation. Dis. Model. Mech..

[34] Dobosz E., Wadowska M., Kaminska M., Wilamowski M., Honarpisheh M., Bryzek D., Potempa J., Jura J., Lech M., Koziel J. (2021). MCPIP-1 Restricts Inflammation via Promoting Apoptosis of Neutrophils. Front. Immunol..

[35] Xiaoming A., Wenbo J., Jinyi W., Bin W., Chunyang H., Qi C., Lianbao K. (2020). Macrophage Regnase-1 Deletion Deteriorates Liver Ischemia/Reperfusion Injury through Regulation of Macrophage Polarization. Front. Physiol..

[36] Wu H., Chen G., Wyburn K.R., Yin J., Bertolino P., Eris J.M., Alexander S.I., Sharland A.F., Chadban S.J. (2007). TLR4 activation mediates kidney ischemia/reperfusion injury. J. Clin. Investig..

[37] Gantier M.P., Tong S., Behlke M.A., Xu D., Phipps S., Foster P.S., Williams B.R.G. (2008). TLR7 is involved in sequence-specific sensing of single-stranded RNAs in human macrophages. J. Immunol..

[38] Vesentini N., Barsanti C., Martino A., Kusmic C., Ripoli A., Rossi A., L’Abbate A. (2012). Selection of reference genes in different myocardial regions of an in vivo ischemia/reperfusion rat model for normalization of antioxidant gene expression. BMC Res. Notes.

[39] Huang S., Miao R., Zhou Z., Wang T., Liu J., Liu G., Chen Y.E., Xin H.-B., Zhang J., Fu M. (2013). MCPIP1 Negatively Regulates Toll-like Receptor 4 Signaling and Protects Mice from LPS-induced Septic Shock. Cell. Signal..

[40] Jin Z., Zheng E., Sareli C., Kolattukudy P.E., Niu J. (2021). Monocyte Chemotactic Protein-Induced Protein 1 (MCPIP-1): A Key Player of Host Defense and Immune Regulation. Front. Immunol..

[41] Kemmner S., Bachmann Q., Steiger S., Lorenz G., Honarpisheh M., Foresto-Neto O., Wang S., Carbajo-Lozoya J., Alt V., Schulte C. (2019). STAT1 regulates macrophage number and phenotype and prevents renal fibrosis after ischemia-reperfusion injury. Am. J. Physiol. Ren. Physiol..

[42] Lorenz G., Moschovaki-Filippidou F., Würf V., Metzger P., Steiger S., Batz F., Carbajo-Lozoya J., Koziel J., Schnurr M., Cohen C.D. (2019). IFN Regulatory Factor 4 Controls Post-ischemic Inflammation and Prevents Chronic Kidney Disease. Front. Immunol..

[43] Satoh T., Takeuchi O., Vandenbon A., Yasuda K., Tanaka Y., Kumagai Y., Miyake T., Matsushita K., Okazaki T., Saitoh T. (2010). The Jmjd3-Irf4 axis regulates M2 macrophage polarization and host responses against helminth infection. Nat. Immunol..

[44] Mittrücker H.W., Matsuyama T., Grossman A., Kündig T.M., Potter J., Shahinian A., Wakeham A., Patterson B., Ohashi P.S., Mak T.W. (1997). Requirement for the transcription factor LSIRF/IRF4 for mature B and T lymphocyte function. Science.

[45] Suzuki S., Honma K., Matsuyama T., Suzuki K., Toriyama K., Akitoyo I., Yamamoto K., Suematsu T., Nakamura M., Yui K. (2004). Critical roles of interferon regulatory factor 4 in CD11bhighCD8alpha- dendritic cell development. Proc. Natl. Acad. Sci. USA.

[46] Vander Lugt B., Khan A.A., Hackney J.A., Agrawal S., Lesch J., Zhou M., Lee W.P., Park S., Xu M., DeVoss J. (2014). Transcriptional programming of dendritic cells for enhanced MHC class II antigen presentation. Nat. Immunol..

[47] Ke X., Chen C., Song Y., Cai Q., Li J., Tang Y., Han X., Qu W., Chen A., Wang H. (2019). Hypoxia modifies the polarization of macrophages and their inflammatory microenvironment, and inhibits malignant behavior in cancer cells. Oncol. Lett..

[48] Bonventre J.V., Yang L. (2011). Cellular pathophysiology of ischemic acute kidney injury. J. Clin. Investig..

[49] Liang J., Wang J., Azfer A., Song W., Tromp G., Kolattukudy P.E., Fu M. (2008). A novel CCCH-zinc finger protein family regulates proinflammatory activation of macrophages. J. Biol. Chem..

[50] Kotlinowski J., Hutsch T., Czyzynska-Cichon I., Wadowska M., Pydyn N., Jasztal A., Kij A., Dobosz E., Lech M., Miekus K. (2021). Deletion of Mcpip1 in Mcpip1fl/flAlbCre mice recapitulates the phenotype of human primary biliary cholangitis. Biochim. Biophys. Acta Mol. Basis Dis..

[51] Günthner R., Kumar V.R.S., Lorenz G., Anders H.-J., Lech M. (2013). Pattern-recognition receptor signaling regulator mRNA expression in humans and mice, and in transient inflammation or progressive fibrosis. Int. J. Mol. Sci..

[52] Steiger S., Kumar S.V., Honarpisheh M., Lorenz G., Günthner R., Romoli S., Gröbmayr R., Susanti H.-E., Potempa J., Koziel J. (2017). Immunomodulatory Molecule IRAK-M Balances Macrophage Polarization and Determines Macrophage Responses during Renal Fibrosis. J. Immunol..

[53] Moschovaki-Filippidou F., Steiger S., Lorenz G., Schmaderer C., Ribeiro A., von Rauchhaupt E., Cohen C.D., Anders H.-J., Lindenmeyer M., Lech M. (2020). Growth Differentiation Factor 15 Ameliorates Anti-Glomerular Basement Membrane Glomerulonephritis in Mice. Int. J. Mol. Sci..

[54] Baek J.-H. (2019). The Impact of Versatile Macrophage Functions on Acute Kidney Injury and Its Outcomes. Front. Physiol..

[55] Ysebaert D.K., De Greef K.E., Vercauteren S.R., Verhulst A., Kockx M., Verpooten G.A., De Broe M.E. (2003). Effect of immunosuppression on damage, leukocyte infiltration, and regeneration after severe warm ischemia/reperfusion renal injury. Kidney Int..

[56] Gonçalves G.M., Cenedeze M.A., Feitoza C.Q., Wang P.M.H., Bertocchi A.P.F., Damião M.J., Pinheiro H.S., Antunes Teixeira V.P., dos Reis M.A., Pacheco-Silva A. (2006). The role of heme oxygenase 1 in rapamycin-induced renal dysfunction after ischemia and reperfusion injury. Kidney Int..

[57] Lieberthal W., Levine J.S. (2009). The role of the mammalian target of rapamycin (mTOR) in renal disease. J. Am. Soc. Nephrol..

[58] Lee S., Huen S., Nishio H., Nishio S., Lee H.K., Choi B.-S., Ruhrberg C., Cantley L.G. (2011). Distinct macrophage phenotypes contribute to kidney injury and repair. J. Am. Soc. Nephrol..

[59] Gordon S., Plüddemann A., Martinez Estrada F. (2014). Macrophage heterogeneity in tissues: Phenotypic diversity and functions. Immunol. Rev..

[60] Kapoor N., Niu J., Saad Y., Kumar S., Sirakova T., Becerra E., Li X., Kolattukudy P.E. (2015). Transcription factors STAT6 and KLF4 implement macrophage polarization via the dual catalytic powers of MCPIP. J. Immunol..

[61] Opal S.M., Scannon P.J., Vincent J.L., White M., Carroll S.F., Palardy J.E., Parejo N.A., Pribble J.P., Lemke J.H. (1999). Relationship between plasma levels of lipopolysaccharide (LPS) and LPS-binding protein in patients with severe sepsis and septic shock. J. Infect. Dis..

[62] Jeltsch K.M., Hu D., Brenner S., Zöller J., Heinz G.A., Nagel D., Vogel K.U., Rehage N., Warth S.C., Edelmann S.L. (2014). Cleavage of roquin and regnase-1 by the paracaspase MALT1 releases their cooperatively repressed targets to promote TH17 differentiation. Nat. Immunol..

[63] Peng H., Ning H., Wang Q., Lu W., Chang Y., Wang T.T., Lai J., Kolattukudy P.E., Hou R., Hoft D.F. (2018). Monocyte chemotactic protein–induced protein 1 controls allergic airway inflammation by suppressing IL-5–producing TH2 cells through the Notch/Gata3 pathway. J. Allergy Clin. Immunol..

[64] Achuthan A., Cook A.D., Lee M.-C., Saleh R., Khiew H.-W., Chang M.W.N., Louis C., Fleetwood A.J., Lacey D.C., Christensen A.D. (2016). Granulocyte macrophage colony-stimulating factor induces CCL17 production via IRF4 to mediate inflammation. J. Clin. Investig..

[65] Lee K.M.-C., Jarnicki A., Achuthan A., Fleetwood A.J., Anderson G.P., Ellson C., Feeney M., Modis L.K., Smith J.E., Hamilton J.A. (2020). CCL17 in Inflammation and Pain. J. Immunol..

[66] Chen Y.-T., Hsu H., Lin C.-C., Pan S.-Y., Liu S.-Y., Wu C.-F., Tsai P.-Z., Liao C.-T., Cheng H.-T., Chiang W.-C. (2020). Inflammatory macrophages switch to CCL17-expressing phenotype and promote peritoneal fibrosis. J. Pathol..

[67] Lee M.-C., Lacey D.C., Fleetwood A.J., Achuthan A., Hamilton J.A., Cook A.D. (2019). GM-CSF- and IRF4-Dependent Signaling Can Regulate Myeloid Cell Numbers and the Macrophage Phenotype during Inflammation. J. Immunol..

[68] Honarpisheh M., Foresto-Neto O., Steiger S., Kraft F., Koehler P., von Rauchhaupt E., Potempa J., Adamowicz K., Koziel J., Lech M. (2018). Aristolochic acid I determine the phenotype and activation of macrophages in acute and chronic kidney disease. Sci. Rep..

[69] Cretney E., Xin A., Shi W., Minnich M., Masson F., Miasari M., Belz G.T., Smyth G.K., Busslinger M., Nutt S.L. (2011). The transcription factors Blimp-1 and IRF4 jointly control the differentiation and function of effector regulatory T cells. Nat. Immunol..

[70] Brüstle A., Heink S., Huber M., Rosenplänter C., Stadelmann C., Yu P., Arpaia E., Mak T.W., Kamradt T., Lohoff M. (2007). The development of inflammatory T(H)-17 cells requires interferon-regulatory factor 4. Nat. Immunol..

[71] Tang P.M.-K., Nikolic-Paterson D.J., Lan H.-Y. (2019). Macrophages: Versatile players in renal inflammation and fibrosis. Nat. Rev. Nephrol..

[72] Sasaki K., Terker A.S., Pan Y., Li Z., Cao S., Wang Y., Niu A., Wang S., Fan X., Zhang M.-Z. (2021). Deletion of Myeloid Interferon Regulatory Factor 4 (Irf4) in Mouse Model Protects against Kidney Fibrosis after Ischemic Injury by Decreased Macrophage Recruitment and Activation. J. Am. Soc. Nephrol..

[73] Liu Z., Zhang J. (2021). Heterogenous Role of IRF4 in Kidney Fibrosis. J. Am. Soc. Nephrol..

